# Bisphenols and Male Reproductive Health: From Toxicological Models to Therapeutic Hypotheses

**DOI:** 10.3389/fendo.2020.00301

**Published:** 2020-06-04

**Authors:** Luca De Toni, Maurizio De Rocco Ponce, Gabriel Cosmin Petre, Kais Rtibi, Andrea Di Nisio, Carlo Foresta

**Affiliations:** ^1^Department of Medicine and Unit of Andrology and Reproductive Medicine, University of Padova, Padova, Italy; ^2^Autònoma de Barcelona, Instituto de Investigaciones Biomédicas Sant Pau, Barcelona, Spain; ^3^Laboratory of Functional Physiology and Valorization of Bioresources, Higher Institute of Biotechnology of Beja, University of Jendouba, Beja, Tunisia

**Keywords:** endocrine discruptors, semen parameters, endocrine axes, drug metabolism, exposure markers

## Abstract

Bisphenols, and in particular bisphenol A (BPA), have been widely used for the production of plastic manufacts in the last 50 years. Currently, BPA is present in a variety of daily use polycarbonate plastics and epoxy resins, and dietary ingestion is considered the main route of human exposure. Accordingly, BPA is the chemical pollutant with the widest exposure in humans, involving nearly 90% of general population, according to recent studies. Concerns about BPA effects on human health date back to 1930s, when severe impact on male sexual development was suggested. Now, the acknowledged biological effects of BPA are various. In regard to human fertility, BPA has been shown to disrupt hormone signaling even at low concentrations. Results from human epidemiological studies have reported BPA interference with follicle stimulating hormone, inhibin B, estradiol, testosterone levels, and sexual function in male subjects. Moreover, recent studies have reported an association between BPA levels and reduced sperm concentration, motility, normal morphology, sperm DNA damage, and altered epigenetic pattern, resulting in trans-generational legacy of BPA effects. In this review, the recognized effects of BPA on male reproductive health are described, from the most recent issues on experimental models to epidemiological data. In addition, the very recent interest about the use of nutraceutical remedies to counteract BPA effects are discussed.

## Introduction

Bisphenols, and in particular the phenol compound 2,2 Bis (4-hydroxylphenyl)–propane, universally known as Bisphenol-A (BPA), are widely used as additives for the production of plastic materials, such as polycarbonate, phenol and epoxy resins, and polyesters and polyacrylates, as well as an antioxidant in foodstuffs and cosmetics (1, 2). Specifically, nearly 75% of the industrial production of BPA is intended for the manufacture of polycarbonate-based products, which find wide application in food industry, such as in containers for food and beverages, in plastic dishes, in kitchen utensils, in containers for microwave cooking, and until 2011, in bottles (3). Of note, BPA is also used in epoxy resin films used as binary patina: the internal coatings in the cans for canned food (4).

BPA is a solid at 25°C with a melting temperature of 156°C, insoluble in water but soluble in alcohol, ethers, and fats. Accordingly, BPA can migrate for continuity in food and drinks by direct contact with plastic container under certain conditions. Prolonged storage times, exposure to high temperatures (e.g., >70°C), and the presence of foods with a significant lipid component represent some of these conditions. Consequently, BPA enters the food chain due to the massive use of plastics, as containers or technological packaging, and as a function of increasing the shelf life of foods (5). A recent report from the European Food Safety Agency (EFSA) showed that the highest concentrations of BPA were found in packaged products (on average 18.68 μg/kg) compared to unpackaged foods (on average 1.50 μg/kg) (6). To this regard, the most relevant concentrations of BPA (>30 μg/kg) were observed in packaged food, such as cereals, meat and fish, ready-to-use foods, snacks, and sweets. As for other bulk foods, the presence of the contaminant is most likely due to the production processes. Among unpackaged foods, the highest concentrations were found in fish, with average values of 9.40 μg/kg. These data strongly suggest a major role of massive plastic pollution in waterways (7).

As a result, there is a significant risk of human exposure to BPA through ingestion, skin contact, or inhalation (8, 9). Once accessed into the body, nearly the 12% of BPA is metabolized in the liver by glucuronidation, providing more water-solubility and quicker excretion in urine, even if the concentrations in plasma and urine are very low and difficult to detect (10, 11). In addition, the conjugated form of BPA is equally accounted in the pool of the active forms (12). For this reason, total urinary BPA, including both conjugated and unconjugated BPA, is generally used as a biomarker of exposure to BPA (13). Epidemiological data from the United States have reported detectable levels of BPA in urine samples from more than 90% of general population, resulting a major problem of exposure to chemical substance (14).

Concerns about BPA issues on the human health date back to 1930s, when severe impact on male sexual development had been suggested. From a mechanistic point of view, the most relevant risks associated with the exposure to BPA are mainly due to its action as an endocrine disruptor (ED), being able to interfere with the balance of the hormonal system and thus causing harmful effects on the whole body (15). Available reports in late 1990s firstly documented a stimulating activity of BPA on estrogen receptor α that differed, however, from the classical pattern observed in weak estrogens, partial agonists, and pure antagonists (16, 17). This evidence was confirmed by subsequent investigations, reporting that BPA binds several nuclear receptors, mimicking the action of endogenous steroids, maintaining the target molecule in active conformations or blocking the access of endogenous 17β-estradiol to receptor's binding site by competition (18–20). In addition, unconjugated BPA showed a binding activity to other two receptors: the G protein-coupled estrogen receptor 30 (GPR30), also known as membrane estrogen receptor alpha (mERα) (21, 22) and the orphan nuclear estrogen-related receptor gamma (ERR-gamma) (23, 24). Finally, experimental animal studies demonstrated that BPA binds also to the androgen receptor (AR), to the peroxisome proliferator-activated receptor gamma (PPAR-gamma), and the thyroid hormone receptor (19).

On these bases, the exposure to BPA is increasingly suspected to exert major reproductive issues, such as the impairment of semen production in men as well as alteration of the hormonal cycle and oocyte maturation in women (25). This narrative review will cover available evidence regarding the male reproductive outcomes associated with the exposure to BPA. In addition, possible remedies to counteract BPA effects are discussed.

## Methods

PubMed, Scopus and Web of Science databases were used to perform a literature search on the time interval 2000–2019. The following terms were included: “bisphenol male fertility,” “bisphenol testis,” “bisphenol reproductive outcome,” “bisphenol semen parameters,” “bisphenol spermatozoa,” “bisphenol nutraceuticals,” “bisphenol dietary supplements,” “bisphenol antioxidant,” “bisphenol medicinal plants.” We included studies on cell models, studies on murine models, and observational studies in humans.

The overall 6,865 records were then screened for relevance to the topics, for a total of 77 studies finally considered for the review. Data from eligible studies were considered separately, according to the different following topics: “data from animal studies,” “data from human studies,” and “nutritional remedies to BPA related disorders.”

### Disrupting Effects of BPA on Male Fertility: Data From Animal Studies

A wide amount of data from animal studies shows a clear effect of BPA on male reproductive system, even at very low doses. One of the first investigations on this topic relied of the fact that BPA is massively used in sealant made of resin-based composite materials for dental use, with the consequent oral ingestion of BPA. Al-Hiyasat et al. were among the first to investigate the reproductive outcome in male mice exposed to BPA by oral ingestion, suggesting possible issues for infertility, genital tract malformations and increased cancer rates in estrogen sensitive target tissues (26). BPA doses >25 ng/kg were associated with reduced sperm count, both at epididymal and ejaculated level, and with significant reductions of the absolute weights of the testes and seminal vesicles. These early results were confirmed by more recent studies reporting decreased sperm count associated with the exposure of BPA in rodent models, suggesting major impairment of the spermatogenetic process. (27–33). In addition, lower levels of exposure were equally associated with reduced semen quality, particularly with regard to motility parameters and markers of adequate cell-redox balance (27, 29, 31, 33, 34). Furthermore, the exposure to BPA has been associated with the alteration of other non-conventional markers of sperm quality such as the index of DNA fragmentation, suggesting a possible role as mutagen (29, 31, 34–42). Also, in a recent study from Wisniewski et al. acrosomal integrity, an overall marker of the fertilization potential, was significantly reduced by PBA exposure in murine models (27).

As outlined by the aforementioned studies, BPA showed major abilities to interfere with spermatogenesis and germ cell maturation, a process largely regulated by the synthesis of testosterone (T) from the Leydig cell population of the testis under the direct control of pituitary luteinizing hormone (LH) (43). Several studies have been performed to disclose the possible disruption of the hypothalamus/hypophysis/testis axis (HHTA) associated with BPA exposure in animal models, with the result of a fairly complex picture that invariably leads to the impaired production of T (28, 44). In this regard, both direct effects on Leydig cells and indirect effects on HHTA were recognized. Among the direct effects, a study conducted in the classical murine Leydig MA-10 cell model, Lan et al. showed that BPA forces a detour of the normal steroidogenic activity by stimulating, on one hand, the production of 17-hydroxy-pregnenolone and T from cholesterol, but on the other hand, the expression of CYP19A1, the aromatase activity that converts T into 17-β estradiol, resulting in a overproduction of this latter (45). Other studies suggested that BPA triggers multi-level dysfunction in Leydig cells, altering either insulin signaling and glucose transport or the mitochondrial activity, with a resulting downstream redox imbalance and altered steroidogenesis (46, 47). As anticipated, BPA was also suggested to indirectly suppress the pituitary LH release through the massive aromatase upregulation in the testes; the consequent increase of serum estrogens would then exert a negative hormonal feedback at central level (48). Importantly, because of its high lipid solubility, BPA undergoes to trans-placental transfer in animal models with a consequent detection in cord blood, an evidence reported also in humans (49–52). Accordingly, BPA exposure during the prenatal period was associated with the impairment of both fetal development and the endocrine function of the testis, with reduced Leydig cell proliferation and fetal testosterone production (53–55). Additional data from animal models suggests that the endocrine disruption associated with BPA exposure in male fetuses negatively affects fertility in adult life. To this regard, in a study by Salian et al., maternal exposure to BPA was associated with reduced sperm count and motility in male offspring and, in turn, with post implantation loss and decreased litter size (56). However, the mechanisms by which BPA interferes with testis development and function, whether in fetal or in adult life, seem to be wider than the exclusive endocrine disruption of the HHTA. In fact, exposure to BPA alters the glucose homeostasis in germ cells through the decreased expression of GLUT-8 glucose transporter, particularly in spermatocytes and spermatids (39). In addition, an increased oxidative stress in the testis was claimed as the responsible for the impaired seminal quality associated with exposure to BPA (35, 57). For example, excessive production of reactive oxygen species (ROS) and consequent mitochondrial dysfunction induced by BPA, was associated with Sertoli cells apoptosis (58). BPA was also suggested to directly interfere with apoptotic signaling and to induce the morphological changes in Sertoli cell mitochondria, the triggering of Pten/Akt signaling pathway, or the activation of the JNKs/p38 MPAK pathway, with the consequent nuclear translocation of NF-kB and Fas/FasL system (59–61). Despite this severe interference with Sertoli cell cycle, a morphological alteration of testicular histologic architecture was not observed frequently, largely depending on the protocol of administration. In fact, Aikawa et al. showed that the experimental exposure of male mice to 50 μg BPA for 5 days after birth caused a decrease in normal morphology and sperm motility with no significant histologic changes of testes (62). Jiang et al. observed ultrastructural lesions in Sertoli and Leydig cells after the administration of 5 mg/kg/day of BPA to rats for 8 weeks (63).

Of note, very recent studies disclosed some transgenerational effects associated with BPA exposure. Manikkam et al. showed that the early exposure of female gestating rats to a cocktail of plastic additives, including BPA, was associated with a significant increase of the prevalence of diseases and abnormalities in F1 and F3 generation males, particularly pubertal abnormalities, testis disease and obesity (64). Likewise, similar effects were exerted by replacement bisphenols, namely compounds structurally similar to BPA used in “BPA-free” products (65). Subsequent studies were able to detect major genetic abnormalities associated with exposure to BPA. Firstly, BPS showed a mutagen effect on male germ cells, resulting in blocked meiotic progression of germ cells (31, 66). Furthermore, Shi et al. showed that both BPA and replacement bisphenols are able to modify the expression of DNA methyltransferases and the pattern of histone methylation in the neonatal and adult testes (67).

However, earlier studies by Hass et al. on this topic (68) reported that male offspring from pregnant Wistar rats, gavaged with bisphenol A from gestation day 7 to pup day 22, showed a significant reduction of the sperm count only at the lowest bisphenol A dose (25 μg/kg/day). Higher doses had no effect on either sperm parameters or the weight and histology of the reproductive organs. These results suggest a likely transgenerational toxicity of bisphenols, with a possible mechanistic involvement of epigenetics on the impairment of male reproductive functions. However, a more complex scenario should be hypothesized given the observed non-monotonic dose–response relationship.

## Disrupting Effects of BPA on Male Fertility: Data From Human Studies

Despite the large availability of data in animal models, fewer studies assessed the possible relationship between BPA exposure and semen quality in humans. The first reports on this topic dealt with occupational medicine; particularly, Li et al. found a negative association between urinary BPA and sperm concentration, total sperm count, viability, and motility in 215 factory workers, further distinguished into occupationally exposed to high or low levels of BPA. However, in the subgroup with lower creatinine-adjusted urinary BPA, the only significant association was with reduced sperm concentration. Notably, urinary BPA levels were not associated to altered morphology in this study (69). As for data in animal models, other studies investigated the possible association of BPA exposure with alterations of sperm DNA. Meeker et al. explored the possible correlation between urinary BPA concentration and sperm DNA damage, evaluated by neutral comet assay, in a cohort of 190 subfertile male patients (70). Urinary BPA concentration was associated with reduced sperm concentration, motility, and morphology, whereas a positive association with sperm DNA damage was observed. However, two independent studies on male partners from infertile couples attending infertility clinics were not able to retrieve any significant association between BPA urinary concentration and altered semen parameters. Importantly, a relatively high variability of exposure markers was observed, since the mean urinary BPA concentration in these two studies were, respectively, 1.5 and 0.6 ng/mL (71, 72).

Another field of investigation pursued was the possible correlation between exposure to BPA and alteration of the endocrine pattern, but widely varying scenarios can be observed. Hanaoka et al. conducted a study on 42 workers occupationally exposed to BPA through the handling of epoxy resin spray containing BPA (73). Interestingly, authors have found lower serum levels of follicle-stimulating hormone (FSH) in exposed workers compared to those non-exposed, although non-obvious differences in plasma LH and free T levels were observed. Also, Galloway et al. investigated the relationship between urinary BPA and male reproductive hormones in a cohort of 715 healthy adults aged 20–74 years from the general population (74). Surprisingly, urinary BPA levels were positively and significantly associated with serum T levels, but no associations with either 17-β estradiol, sex hormone-binding globulin (SHBG), or free T were observed. On the other hand, Lassen et al., in a study on 308 healthy males from the general population, found increased serum T, free T, LH, and estradiol in subjects pertaining to higher urinary BPA concentrations quartile, compared with the lowest quartile. Subjects in the highest urinary BPA quartile also showed reduced progressive sperm motility compared with the lowest quartile (75). Also, Mendiola et al. performed a similar study on 375 fertile men recruited from prenatal clinics, finding that urinary BPA concentrations were positively associated with serum SHBG levels and inversely correlated with free androgen index (FAI), calculated as total T × 100/SHBG and the FAI/LH ratio. However, serum FSH, LH, total T, inhibin B, and free T levels showed no obvious correlation with urinary BPA concentration (72). In addition, Meeker et al. found a negative association between urinary BPA levels and both serum inhibin B levels and 17-β estradiol/T ratio in male partners of subfertile couples attending a fertility clinic; however, BPA was positively associated with both FSH and FSH/inhibin B ratio (76).

Finally, few studies aimed to assess the possible impact of BPA exposure on the overall fertility potential in males through the overall evaluation of the relationship between BPA levels and the reproductive outcome in the setting of assisted reproduction facilities. In a study enrolling 215 infertile couples undergoing assisted reproduction techniques, with roughly equal distribution between *in vitro* fertilization and intrauterine insemination, Dodge et al. (77) investigated the possible correlation between urinary concentrations of parabens and BPA with the live-birth rate. Authors found minimal association between paternal urinary propyl paraben levels and reduced live-birth rate in a correlation model corrected by possible confounders. However, no significant association emerged between paternal urinary BPA and reproductive outcomes after fertility treatments (77). On the other hand, Buck-Louis et al. in the Longitudinal Investigation of Fertility and the Environment (LIFE) Study, a multicenter investigation involving 501 infertile couples from 16 targeted counties in the middle-east of the United States (78), evaluated the possible relationship between time to pregnancy (TTP) and urine levels of more than 15 environmental pollutants, including BPA, in both males and females. Urinary BPA concentration in either males or females was not associated with increased TTP, which was instead correlated with male urinary concentration of monomethyl, mono-n-butyl, and monobenzyl phthalates.

Overall, available data are supportive of detrimental role of BPA on semen parameters, but this is not accompanied by clear data on sex hormones and on fertility outcomes. As suggested by other authors (79), within the limits of the availability of data in humans, a possible reconciling explanation could rely on a greater direct toxicity of BPA on germ line cells, rather than in an albeit important endocrine disruption of the HHTA. This hypothesis is somewhat supported by very few studies reporting the interference of BPA on germ cell development in human fetal testis and on mitochondrial activity and energy metabolism in ejaculated human sperms (57, 80, 81).

## Nutraceutical Approaches to Overcome BPA Effects

Given the large availability of evidence reporting detrimental effects of BPA on testis function, especially in animal models, this chemical has progressively gained a role as a reference substance, able to induce endocrine disruption in several experimental models, from laboratory animals to *in vitro* cell cultures (82). On this basis, some recent studies have focused on possible approaches to treat or prevent BPA-induced derangements and testicular toxicity. Since the direct toxicodynamics of PBA on both Leydig and germ cells of the testis were largely related to the impairment of cell redox system, most of the treatment approaches relied on the use of natural sources of antioxidants.

Based on the fact that the expression of the enzymes glutathione peroxidase and glutathione reductase are regulated by melatonin, a study from Anjum et al. aimed to disclose the possible effect of melatonin on mitochondrial lipid peroxidation observed in mouse testis after BPA exposure (41). Interestingly, the treatment with melatonin reduced mitochondrial lipid peroxidation, restored the overall mitochondrial enzyme machinery and improved the mitochondrial antioxidant pool compromised by BPA. However, major limitations of the study were represented by the high dosage of melatonin administered intraperitoneally. In 2012, El-Beshbishy et al. demonstrated some mitigation of the mitochondrial toxicity exerted by BPA exposure in rats, by the co-administration of lipoic acid (44). Also, Khalaf et al. (83) recently reported a protective effect of selenium (Se) against BPA-induced testis impairment in albino male rats. In particular, co-administration of Se attenuated the reproductive issues induced by BPA toxicity through the restoration of testicular antioxidant activity and the amelioration of sperm genetic abnormalities observed in BPA exposed animals (83). Similar results were obtained by Kaur et al. who reported decreased lipid peroxidation in mouse testis associated with the co-administration of Se and BPA, compared with sole exposure to BPA (84).

Another key vitamin supplementation, namely vitamin D, showed a partial restore of testicular fibrosis in a complex rat model of diabetes, obtained by streptozotocin treatment, associated with BPA-induced hypogonadism (85). Interestingly, this effect appeared as the result of a direct downregulation of nuclear factor kappa B exerted by vitamin D, rather than the indirect involvement of the central pituitary/testis axis.

Of note, the composition of the antioxidant mixture seems to have major relevance on the efficacy of the treatment. In fact, the classical vitamin C administration failed to produce any amelioration on the testicular oxidative damage induced by BPA in rats, or even exerted worsening effects (86). On the contrary, Rahman et al. (87), in an *in vitro* experimental model on isolated mouse spermatozoa, showed that the combination of glutatione, vitamin C, and vitamin E effectively prevented the oxidative stress and the respective downstream tyrosine phosphorylation-signaling pathway, avoiding the premature acrosome reaction and possibly improving the fertilization capacity of sperm cells exposed to BPA. (87).

Interestingly, a wide variety of phytochemicals and plant extracts showed ameliorating effects of testis function in rodent models exposed to BPA (88). *Cordyceps militaris*, a medical fungus largely employed in Chinese traditional medicine, restored the histological architecture of seminiferous tubules and epididymis in male rats exposed to BPA, with a significant recovery of the sperm count, through the likely reduction of the oxidative stress damage (89). Also, lycopene showed a detoxifying activity toward testicular damages associated with BPA exposure, as evidenced by the protection from the loss of germ cell population, the reduction of testis and epididymis weight, as well as the impairment of sperm motility, exerted by the treatment of male rats with the sole BPA (90). Furthermore, co-administration of quercetin, an antioxidant phytochemical member of the polyphenolic flavonoid family, amended the toxic effects on testis and epididymis exerted by BPA (91).

Our group recently showed that the metabolic/mitochondrial disruption, induced by the *in vitro* exposure of human spermatozoa to BPA, was effectively compensated by low dose treatment with aqueous extract from leaves of *Eruca sativa*, a plant of the Brassicaceae family widely represented in the Mediterranean region. Importantly, the characterization of the extract showed to be extremely rich in natural antioxidants, such as polyphenols and flavonoids. The treatment with high concentration of the aqueous extract was unexpectedly associated with severe disruption of both mitochondrial and cell membrane redox balance, resulting in a significant loss of sperm motility (81). Importantly, these preliminary results have been confirmed by a subsequent study performed on Wistar rats (92). Consistent with *in vitro* data, the overall hormonal and semen disruption associated with BPA exposure was significantly ameliorated by the low-dose administration of *Eruca sativa* aqueous extract, while it was worsened by high dose treatment.

Despite these encouraging results, exogenous antioxidants may exert a double-edged effect. In particular, the SELECT study found that the supplementation of vitamin E significantly increased the risk of prostate cancer among healthy men (93). Furthermore, more recently, it has been shown that vitamin E can act as pro-oxidant agent promoting DNA damage and cell transformation (94). Thus, the use of antioxidants-based dietary supplements for the prevention of disease states in general, and in particular for the compensation from altered states associated with exposure to environmental factors, should be considered with caution.

## Conclusions and Future Perspectives

Bisphenol A represents one of the most controversial chemical pollutants, with the typical features of an endocrine disruptor. Early toxicological evidence on BPA date back to nearly 30 years ago, when major interference with estrogen signaling pathway was claimed. Since that time, a wide range of cell mechanisms of both endocrine and metabolic disruption have been claimed by the use of experimental models. In particular, major impairment of the male hypothalamus/hypophysis/testis axis has been recognized as associated with the exposure to BPA during both the fetal and the adult life, resulting in altered testis development, impaired endocrine function and infertility. In this regard, direct disruption of sperm characteristics, such as reduced motility performances and development genetic abnormalities have been identified. On the other hand, data obtained in humans are actually limited and poorly conclusive to identify a strict causal role of BPA in reduced male fertility potential. A summary of references supporting each singular effects of bisphenols on male reproductive health is reported in Table 1.

**Table 1:** Summary of the references supporting the possible effects of bisphenols on male reproductive health.

	**Animal Model**		**Human Model**	
**Outcome**	***In vitro***	***In vivo***	***In vitro***	***In vivo***
Testis Histology		(62) **↔** (63) **↓**		
Effect on sperm count		(26) **↓** (29) **↓** (28) **↓** (61) **↓** (**↓**Sertoli cell function) (32) **↓** (33) **↓** (30) **↓** (31) **↓** (59) **↓** (**↓**Sertoli cell function) (60) **↓** (**↓**Sertoli cell function) (27) **↓**		(71) **↔** (69) **↓↔** (95) **↓** (72) **↔**
Effect on sperm motility/mitochondrial function		(39) **↓** (**↓** Germ/Sertoli cells metabolism) (29) **↓** (34) **↓** (33) **↓** (31) **↓** (27) **↓**	(57) **↓** (35) **↓** (80) **↓** (81) **↓**	(71) **↔** (75) **↓** (95) **↓** (72) **↔**
Sperm DNA Fragmentation		(41) **↑** (35) **↑** (39) **↑** (29) **↑** (42) **↑** (40) **↑** (36) **↑** (34) **↑** (37) **↑** (31) **↑** (38) **↑**		(70) **↑**
Testosterone Production	(46) **↓** (**↓** redox balance) (47) **↓** (**↓** redox balance) (45) **↓** (**↑** CYP19)	(53) **↓** (**↓**Fetal testis development) (44) **↓** (28) **↓** (55) **↓** (**↓**Fetal testis development) (54) **↓** (**↓**Fetal testis development) (48) **↓** (**↓** LH by estrogens)		(74) **↑** (73) **↔** (75) **↑** (76) **↓** (72) **↓↔**
Fertility Outcome				(78) **↔** (77) **↔**
Fertility in Offspring		(66) **↓** (68) **↓↔** (64) **↓** (56) **↓** (**↓** Fetal testis development) (67) **↓** (31) **↓**		

*For each outcome considered, the respective references are listed according to the model used, animal or human, the in vitro or in vivo evidence and the observed effect (↓, decrease; ↑, increase; ↔↓, mild decrease or no effect). When available, mechanistic details are provided*.

Methodological differences and different study populations are factors that can explain some discrepancies. Moreover, available clinical outcomes, such as semen parameters and time to pregnancy, are likely susceptible of variation related to many different confounding factors. It should be noted that, as for most of chemical pollutants, the identification of a reliable marker of exposure remains a major issue. Specifically, for BPA, urinary concentrations are surely reliable data from an analytical point of view, but may not be representative of the real exposure to BPA due to its short half-life. To this regard, Vitku et al. reported that BPA levels in blood plasma were positively correlated with BPA levels in semen, but only seminal BPA was negatively associated with seminal quality (96). Finally, the cross-sectional design of the available studies surely provides proof of association, but limited evidence of causality.

One of the main problems associated with exposure to endocrine disruptors in general, and to BPA in particular, is represented by the potential activity at low concentrations. This represents a critical issue during the development phases, such as embryo/fetal life and newborn or peri-pubertal age, since the effects in these time windows may be irreversible and are generally detected only at adulthood (15). Accordingly, populations at higher risk includes pregnant women, infants, and adolescents. On these bases, the current European law restricted the use of BPA in the production of packaging and materials in direct contact with food by limiting migration rate to 0.05 mg/kg of food and prescribing the total absence in products for newborns, from food to food containers and clothes (6). In addition, based on new toxicological data and methodologies, the European Authorities adjusted the tolerable daily intake from 50 to 4 μg/kg body weight/day with an overall lowering rate of 12 times, highlighting the increasing level of attention for these health concerns.

In conclusion, reproductive issues associated with bisphenol A exposure still remains an intense field of investigation, particularly dealing with health consequence reported in males. Current challenges for the future are represented by the identification of efficient markers of exposure in order to address the extent of health consequences in different age ranges.

## Author Contributions

CF supervised manuscript drafting. MD reviewed clinical data. GP and KR reviewed data on nutraceuticals. AD reviewed experimental data. LD drafted the manuscript.

## Conflict of Interest

The authors declare that the research was conducted in the absence of any commercial or financial relationships that could be construed as a potential conflict of interest. The reviewer GR declared a past co-authorship with one of the authors MD to the handling editor.
